# Editorial: The impact of climate and environmental change on epigenetics and pediatric health

**DOI:** 10.3389/fped.2025.1724284

**Published:** 2025-11-19

**Authors:** Erin Kristin Zinkhan

**Affiliations:** 1 Department of Pediatrics, The University of Utah, Salt Lake City, UT, United States; 2 Department of Pediatrics, Intermountain Health, Salt Lake City, UT, United States

**Keywords:** environmental change, climate change, greenness, pediatric health, communicable disease

Modulation of gene expression allows adaptation to environmental changes. Epigenetic mechanisms contribute to both acute and persistent gene regulation and are hypothesized to be a primary way in which an organism can adapt to the changing environment. Climate and environmental change is associated with rapid alterations in critical factors such as air and water quality, access to healthy food, and safe living environments. Altered access to these resources changes the organisms' physiology and epigenetic profile for subsequent generations, impacting the ability of future generations to respond to ongoing climate change.

In humans, climate variability displaces an increasing number of people from their ancestral homes, inducing stressors not only on the displaced but also to the system to which they migrate. Shifting of preferred vector species' environments introduces diseases such as dengue fever and Lyme disease into previously unaffected human populations. Pregnant women, fetuses, newborns, and children are often disproportionately impacted by diseases affected by climate and environmental change. Furthermore, increased exposure to pollution and urbanization and decreased exposure to green spaces impacts both mental and physical health, particularly among children.

Within this research topic, five articles have been published that advance our knowledge about the impact of environmental change on pediatric health.

Two of the five manuscripts in this Research Topic focus on the cognitive and behavioral impact of environmental change. Liu et al. explored the combined exposure to fine particulate matter and greenness during developmentally sensitive periods of time on the development of autism spectrum disorders. The combination of elevated PM2.5 and low greenness increased the risk of developing autism spectrum disorders. However, increased exposure to greenness during the first six months after birth could mitigate these findings. Yangzong et al. evaluated cognitive function in Tibetan adolescents as a factor of education and urbanization and as influenced by altitude. The authors found that high altitude may negatively affect cognition in adolescents, an effect which was reduced by moving to lower altitude and increased years of formal education.

Three of the five manuscripts in this collection focus on the risk of infection as influenced by environmental change. Li et al. evaluated the association between meteorological factors and urinary tract infection in children. Authors found an increase in the number of pathogenic bacteria in pediatric urine culture samples as a factor of average monthly temperature, precipitation, and daylight hours, potentially due to environmental factor influence on survival of pathogenic bacteria and increased pathogenic bacteria in urban streams. Gündüz et al. evaluated the impact of the Coronovirus-19 pandemic on rotavirus infections in children. Authors found more emergency department visits for rotavirus infections after the start of the Coronovirus-19 pandemic, which may have been from a change in healthcare-seeking behavior. Finally, Liu et al. evaluated the influence of environmental factors on hemorrhagic fever with renal syndrome. Authors found a significant correlation between precipitation, temperature, humidity, and daylight hours and hemorrhagic fever with renal syndrome cases, most likely as a result of the influence of these environmental factors on rodent populations and their interaction with human populations.

In conclusion, environmental factors impact multiple aspects of pediatric health. Further research is needed to delineate the impact on the microbiome, social aspects of health, and epigenetic alterations that occur as a result of environmental change, changes in disease patterns, and human migration.

